# Connective tissue growth factor mediates bone morphogenetic protein 2-induced increase in hyaluronan production in luteinized human granulosa cells

**DOI:** 10.1186/s12958-022-00937-y

**Published:** 2022-04-08

**Authors:** Hsun-Ming Chang, Long Bai, Yi-Min Zhu, Peter C. K. Leung

**Affiliations:** 1 Department of Reproductive Endocrinology, Women’s Hospital, School of Medicine, Zhejiang University, Hangzhou, 310006 Zhejiang China; 2 Reproductive Medicine Center, Department of Obstetrics and Gynecology, China Medical University Hospital, Taichung, Taiwan; 3 Department of Obstetrics and Gynaecology, University of British Columbia, and BC Children’s Hospital Research Institute, Room 317, 950 West 28th Avenue, Vancouver, BC V5Z 4H4 Canada

**Keywords:** BMP2, CTGF, HAS2, Hyaluronan, Human granulosa cells

## Abstract

**Background:**

Hyaluronan is the main component of the cumulus-oocyte complex (COC) matrix, and it maintains the basic structure of the COC during ovulation. As a member of the transforming growth factor β (TGF-β) superfamily, bone morphogenetic protein 2 (BMP2) has been identified as a critical regulator of mammalian folliculogenesis and ovulation. However, whether BMP2 can regulate the production of hyaluronan in human granulosa cells has never been elucidated.

**Methods:**

In the present study, we investigated the effect of BMP2 on the production of hyaluronan and the underlying molecular mechanism using both immortalized (SVOG) and primary human granulosa-lutein (hGL) cells. The expression of three hyaluronan synthases (including HAS1, HAS2 and HAS3) were examined following cell incubation with BMP2 at different concentrations. The concentrations of the hyaluronan cell culture medium were determined by enzyme-linked immunosorbent assay (ELISA). The TGF-β type I receptor inhibitors (dorsomorphin and DMH-1) and small interfering RNAs targeting ALK2, ALK3, ALK6 and SMAD4 were used to investigate the involvement of TGF-β type I receptor and SMAD-dependent pathway.

**Results:**

Our results showed that BMP2 treatment significantly increased the production of hyaluronan by upregulating the expression of hyaluronan synthase 2 (HAS2). In addition, BMP2 upregulates the expression of connective tissue growth factor (CTGF), which subsequently mediates the BMP2-induced increases in HAS2 expression and hyaluronan production because overexpression of CTGF enhances, whereas knockdown of CTGF reverses, these effects. Notably, using kinase inhibitor- and siRNA-mediated knockdown approaches, we demonstrated that the inductive effect of BMP2 on the upregulation of CTGF is mediated by the ALK2/ALK3-mediated SMAD-dependent signaling pathway.

**Conclusions:**

Our findings provide new insight into the molecular mechanism by which BMP2 promotes the production of hyaluronan in human granulosa cells.

## Introduction

During the ovulation phase, the intrafollicular components undergo multiple structural changes, including the extracellular matrix remodeling and cumulus-oocyte complex (COC) expansion [1]. The process of COC expansion is initiated by the massive production of granulosa cell-derived hyaluronan, which constitutes the principal structural backbone of COC. Subsequently, several related proteins are produced and attached to this backbone structure, which is essential for COC expansion [2]. To date, three hyaluronan synthases (including HAS1, HAS2 and HAS3) have been identified in mammals [3, 4]. Even though these three enzymes share a similar structure, they are encoded by different genes located on different chromosomes and have different catalytic properties [3, 4]. In rats, hyaluronan synthases are mainly expressed in the theca and granulosa cells of growing follicles [5]. Targeted deletion of *Has2* in mice exhibits embryonic lethality due to developmental deficiency in the cardiovascular system, whereas double knockout of *Has1* and *Has3* in mice is viable and fertile [6, 7]. The expression of these hyaluronan synthases is regulated in a tissue- and agonist-specific manner [8, 9]. For instance, the expression of HAS2 is relatively high during the process of ovulation. Specifically, the administration of hCG significantly increases the expression of HAS2 in granulosa and cumulus cells [10–13]. Interestingly, data obtained from clinical studies indicate that the transcript levels of HAS2 in human cumulus cells are positively correlated with oocyte developmental competence and fertilization ability [14, 15]. Given the essential role of hyaluronan in the regulation of oocyte maturation and ovulation, studies regarding the underlying mechanisms by which various factors regulate the biosynthesis of hyaluronan will help with understanding the regulatory network in folliculogenesis.

Connective tissue growth factor (CTGF, also known as CCN2) is a member of the CCN protein family that plays a crucial role in regulating the growth and differentiation of various cells [16, 17]. CTGF is highly expressed in the female reproductive system and acts as a mediator to regulate reproductive function [18, 19]. Previous in vivo studies have shown that CTGF is mainly expressed in granulosa cells but not in thecal cells [20–22]. The concentration of CTGF in follicular fluid increases during follicle development and reaches a peak level during ovulation [20, 22]. *Ctgf*-deficient mice exhibit severe subfertility and multiple reproductive defects, indicating that CTGF is an essential regulator of follicle development and ovulation [23].

As a member of the transforming growth factor-β (TGF-β) superfamily, bone morphogenetic protein 2 (BMP2) has been shown to participate in the regulation of multiple ovarian functions, including follicle development, steroidogenesis, corpus luteum formation and luteolysis [24–27]. In hamster ovaries, BMP2 is an activator that promotes primordial follicle formation by inducing the transition from germ cells to oocytes [25]. Prior to dominant follicle selection, BMP2 maintains granulosa cells in the undifferentiated state by inhibiting the expression of FSH receptor (FSHR) in hen granulosa cells [28]. BMP2 also upregulates the expression of FSHR and aromatase but downregulates the expression of the LH receptor (LHR) and steroidogenic acute regulatory protein (StAR) in human granulosa cells [26]. Our previous studies have shown that BMP2 prevents premature luteinization by suppressing the expression of PTX3 in human granulosa cells [27]. During the ovulation stage, BMP2 promotes COC expansion by promoting lysyl oxidase activity in human granulosa-lutein (hGL) cells [29]. Moreover, BMP2 is a potent mediator of luteolysis during the luteal phase [24, 30]. Clinical studies have also shown that BMP2 is a potential predictor of oocyte quality during assisted reproductive techniques [31]. Taken together, these findings indicate that BMP2 plays a critical role in the regulation of ovarian function in humans.

Given the important roles of these three intrafollicular factors (hyaluronan, CTGF and BMP2) in the regulation of COC expansion, we aimed to investigate the effect of BMP2 on the production of hyaluronan and the involvement of CTGF in the underlying mechanisms on hGL cells.

## Materials and methods

### Preparation of primary hGL and SVOG cells culture

All of the experiments in this study were approved by the University of British Columbia Research Ethics Board and the Institutional Review Board. Primary hGL cells were isolated from follicular fluid samples of in vitro fertilization (IVF) patients with informed consent and purified by Ficoll Paque density gradient centrifugation as previously described [27, 32]. To reduce the variance of the primary granulosa-lutein cells, primary granulosa-lutein cells derived from human follicular fluid were obtained from women undergoing IVF treatment because of tubal obstruction or male factors. All women recruited to this study have similar age, regular menstrual cycle, and normal ovarian function, and they received the same controlled ovarian hyperstimulation protocol. An immortalized human granulosa-lutein cell line (SVOG) was also utilized. The SVOG cell line was immortalized by the SV40 large T antigen, which was transfected into primary hGL cells obtained from women undergoing an IVF procedure [33]. Both the primary hGL and SVOG cells were seeded into 6-well plates at a density of 2 × 10^5^ cells/well and cultured in Dulbecco’s modified Eagle medium (DMEM)/nutrient mixture F-12 Ham (DMEM/F-12; Sigma–Aldrich Corp., Oakville, ON) supplemented with 10% fetal bovine serum (HyClone, Logan, UT), 100 U/mL penicillin and 100 mg/mL streptomycin sulfate (Life Technologies, Inc./BRL, Grand Island, NY and 1 × GlutaMAX (Life Technologies). The cell culture conditions were in a humidified atmosphere of 5% CO_2_ and 95% air at 37 °C. The cell culture medium was changed every 2 days in all experiments.

### Antibodies and reagents

The polyclonal anti-SMAD4 (9515) antibody was purchased from Cell Signaling Technology (Danvers, MA). Monoclonal anti-α-tubulin (sc-23,948) and polyclonal anti-CTGF (sc-14,939) antibodies were obtained from Santa Cruz Biotechnology (Santa Cruz, CA). Horseradish peroxidase (HRP)-conjugated goat anti-mouse and goat anti-rabbit immunoglobulin G were obtained from Bio-Rad Laboratories (Hercules, CA). HRP-conjugated donkey anti-goat immunoglobulin G was obtained from Santa Cruz Biotechnology. Recombinant human BMP2, dorsomorphin dihydrochloride (dorsomorphin), and dorsomorphin homolog 1 (DMH-1; 4-[6-[4-(1-methylethoxy) phenyl] pyrazole [1, 5-a] pyrimidin-3-yl]-quinoline) were obtained from R&D Systems (Minneapolis, MN). CTGF overexpression and CONTROL vectors were obtained from Gene Copoeia (Rockville, MD). In this study, two specific protein kinase inhibitors DMH-1 (an inhibitor of ALK2/3) and dorsomorphin (an inhibitor of ALK2/3/6) were used to conduct the loss of function experiment by blocking the downstream signaling pathways of ALK2/36. Specifically, the cells were pretreated with DMH-1 (0.25 μM) or dorsomorphin (10 μM) for 1 h, and cells were then treated with a vehicle control or 50 ng/ml BMP2 for 6 h (for examining mRNA levels) or 12 h (for examining protein levels).

### Small interfering RNA transfection and overexpression of CTGF

To knock down endogenous SMAD4, CTGF, ALK2, ALK3 and ALK6, cells were cultured to approximately 70% density and transfected with 25 nM ON-TARGET plus SMART pool small interfering RNA (siRNA) that targets specific genes or siControl nontargeting pool siRNA as the transfection control (Dharmacon, Lafayette, CO) using Lipofectamine RNA iMAX according to the manufacturer’s instructions (Invitrogen/Life Technologies, Burlington, ON, Canada). The knockdown efficiency of the target siRNA was detected by reverse transcription quantitative real-time polymerase chain reaction (RT-qPCR) or western blot analysis. CTGF overexpression (1 μg) or control plasmids were transfected into the cells using Lipofectamine 3000 (Invitrogen, Life Technologies). The overexpression efficiency of CTGF was detected by RT-qPCR analysis.

### Reverse transcription quantitative real-time polymerase chain reaction

Total RNA was extracted with TRIzol reagent (Invitrogen, Life Technologies) according to the manufacturer’s instructions. A total of 2 μg RNA was reverse transcribed into first-strand complementary DNA (cDNA) with random primers and Moloney murine leukemia virus reverse transcription (Promega, Madison, WI). Each 20 μL quantitative polymerase chain reaction sample contained 10 mL of 2 × SYBR Green PCR Master Mix (Applied Biosystems), 20 ng of cDNA, and 250 nM of each specific primer. The specific primers used in the RT-qPCR were as follows: *HAS1* (NM_001523.4): 5′- CAAGATTCTTCAGTCTGGAC − 3′ (sense) and 5′- TAAGAACGAGGAGAAAGCAG − 3′ (antisense); *HAS2* (NM_005328.3): 5′- GCCTCATCTGTGGAGATGGT − 3′ (sense) and 5′- TCCCAGAGGTCCACTAATGC − 3′ (antisense); *HAS3* (NM_005329.3): 5′- CTTAAGGGTTGCTTGCTTGC − 3′ (sense) and 5′- GTTCGTGGGAGATGAAGGAA − 3′ (antisense); *CTGF* (NM_001901.4): 5′- GCGTGTGCACCGCCAAAGAT − 3′ (sense) and 5′- CAGGGCTGGGCAGACGAACG − 3′ (antisense); glyceraldehyde- 3-phosphate dehydrogenase (*GAPDH*) (NM_002046.7), 5′-GAGTCAACGGATTTGGTCGT-3′ (sense) and 5′-GACAAGCTTCCCGTTCTCAG-3′ (antisense). Each cDNA sample was calculated by obtaining the mean value of the triplicate measurements from PCR. The relative quantification of the mRNA levels was performed using the comparative cycle threshold (Ct) method with the 2^-△△Ct^ value formula, and GAPDH was used as the internal reference gene. All primers used in this study passed the validation test.

### Western blot analysis

Cells were lysed in cell lysis buffer (Cell Signaling Technology), and the total protein concentration was measured by a Pierce Rapid Gold BCA kit according to the manufacturer’s instructions (Thermo Fisher, Waltham, MA). Equal amounts of protein were loaded onto polyacrylamide gels, separated by sodium dodecyl sulfate–polyacrylamide gel electrophoresis and then transferred onto polyvinylidene difluoride membranes. After that, the membranes were blocked with Tris-buffered saline (TBS) containing 5% nonfat dry milk for 1 h and incubated overnight at 4 °C with primary antibodies. The next day, the membranes were washed with TBS 10 min each time for a total of six times and then incubated with the appropriate HRP-conjugated secondary antibody for 30 min. Similarly, the membranes were washed with TBS for 10 min for a total of six times after secondary antibody incubation. Finally, the immunoreactive bands were detected with an enhanced chemiluminescent substrate and X-ray film. The intensities of the bands were quantified with Image-Pro Plus software (v4.5; Media Cybernetics, Carlsbad, CA).

### Measurement of hyaluronan and CTGF production

Conditional cell culture medium after treatment was collected and either analyzed immediately or stored at − 80 °C until analysis. The concentrations of the hyaluronan and CTGF in cell culture medium were determined by enzyme-linked immunosorbent assay (ELISA) according to the manufacturer’s instructions (R&D Systems and LifeSpan BioSciences, respectively). The hyaluronan and CTGF levels were normalized to the protein concentration of each cell lysate. The inter- and intra-assay coefficients of variation for both assays were less than 6%. The detection limit of hyaluronan ranged from 0.6 to 40 ng/ml. The detection limit of CTGF ranged from 62.5 to 4000 pg/ml.

### Statistical analysis

The data are presented as the mean ± SEM of at least three independent experiments. PRISM 6.0 software (GraphPad Software Inc., San Diego, CA) was used to perform a one-way analysis of variance followed by Duncan’s test for multiple comparisons of means. Letters are used to denote the statistically significant differences between variables. If the letters on the column bar of two groups are the same (E.g. “a” vs. “a” or “b” vs. “b”), it means that there is no statistical difference between two groups. On the other hand, if the letters on the column bar of two groups are different (E.g. “a” vs. “b” or “b” vs. “c”), it means that there is significant difference between two groups. Meanwhile, for experiments involving only two groups, the data were analyzed by Excel with a two-sample *t*-test assuming unequal variances. *P* < 0.05 was considered statistically significant.

## Results

### BMP2 increases the expression of HAS2 and the production of hyaluronan in hGL cells

**Fig. 1:**
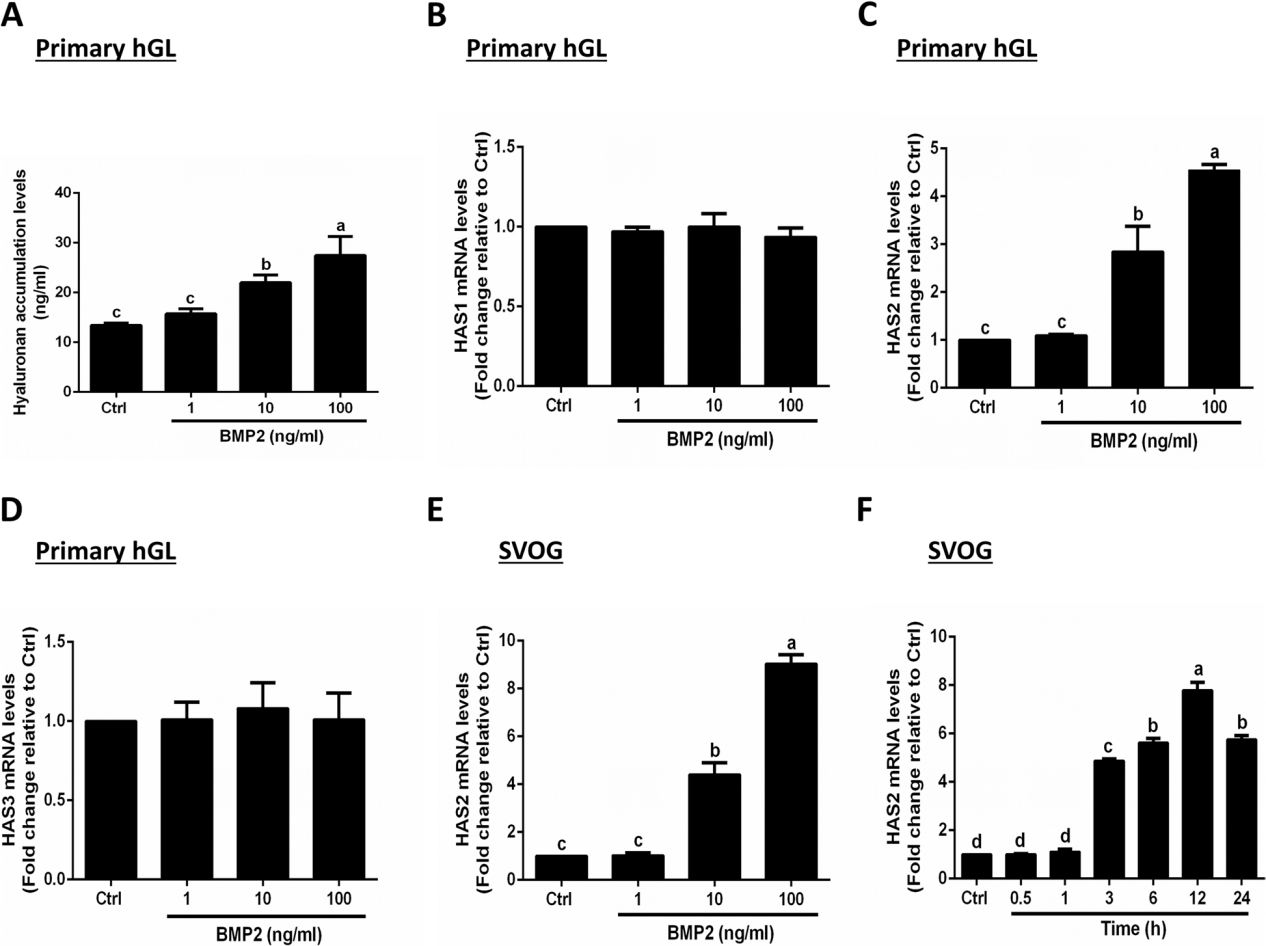
Effects of BMP2 on the accumulated levels of hyaluronan and the expression of hyaluronan synthases in human granulosa-lutein (hGL) cells. **A-D** Primary hGL cells were treated with a vehicle control (Ctrl) or different concentrations (1, 10, or 100 ng/ml) of BMP2 for 24 h. The accumulated levels of hyaluronan in conditioned medium were examined using enzyme-linked immunosorbent assay (ELISA) (**A**), and the mRNA levels of three hyaluronan synthases (HAS1, HAS2 and HAS3) were examined using RT-qPCR (**B-D**). **E** SVOG cells were treated with Ctrl or different concentrations (1, 10, or 100 ng/ml) of BMP2 for 24 h, and the mRNA levels of HAS2 were examined using RT-qPCR. **F**, SVOG cells were treated with Ctrl or BMP2 (50 ng/ml) at different time points (0.5, 1, 3, 6, 12 or 24 h), and the mRNA levels of HAS2 were examined using RT-qPCR. The results are expressed as the mean ± SEM of at least 3 independent experiments, and values without common letters are significantly different (*P* < 0.05). Letters are used to denote the statistically significant differences between variables. If the letters on the column bar of two groups are the same (E.g. “a” vs. “a” or “b” vs. “b”), it means that there is no statistical difference between two groups. On the other hand, if the letters on the column bar of two groups are different (E.g. “a” vs. “b” or “b” vs. “c”), it means that there is significant difference between two groups. BMP2, bone morphogenetic protein 2; Ctrl, control; h, hour

To investigate the effect of granulosa cell-derived BMP2 on the production of hyaluronan, primary hGL cells were treated with a vehicle control or different concentrations of BMP2 (1, 10 or 100 ng/ml) for 24 h. The concentrations of BMP2 used in this study were based on a clinical study showing that the concentrations of BMP2 in human follicular fluid ranged from 1 to 115 ng/ml (with average levels of 45-55 ng/ml) [31]. Using ELISA analysis, the results showed that BMP2 increased the production of hyaluronan in a dose-dependent manner (Fig. 1A). Specifically, 10 and 100 ng/ml BMP2 significantly increased hyaluronan production, while 1 ng/ml of BMP2 did not have this effect (Fig. 1A). Next, we further examined the expression level changes of the three hyaluronan synthases after BMP2 treatment. Using RT-qPCR, our results showed that BMP2 significantly upregulated the expression of HAS2, but not HAS1 and HAS3 in primary hGL cells, indicating that HAS2 is the main hyaluronan synthase involved in the BMP2-induced increase in hyaluronan production (Fig. 1D). In addition, similar effect occurred in SVOG cells showing that BMP2 also upregulated the expression of HAS2 in a concentration-dependent manner (Fig. 1E). To mimic the physiological role of BMP2 on granulosa cell function, we thus chose the concentration of 50 ng/ml BMP2 to challenge the cells in the following experiments. The time course study showed that BMP2 (50 ng/ml) increased the mRNA levels of HAS2, starting at 3 h after treatment, and the effect persisted until 24 h (Fig. 1F).

### BMP2 upregulates the expression of CTGF in hGL cells

**Fig. 2:**
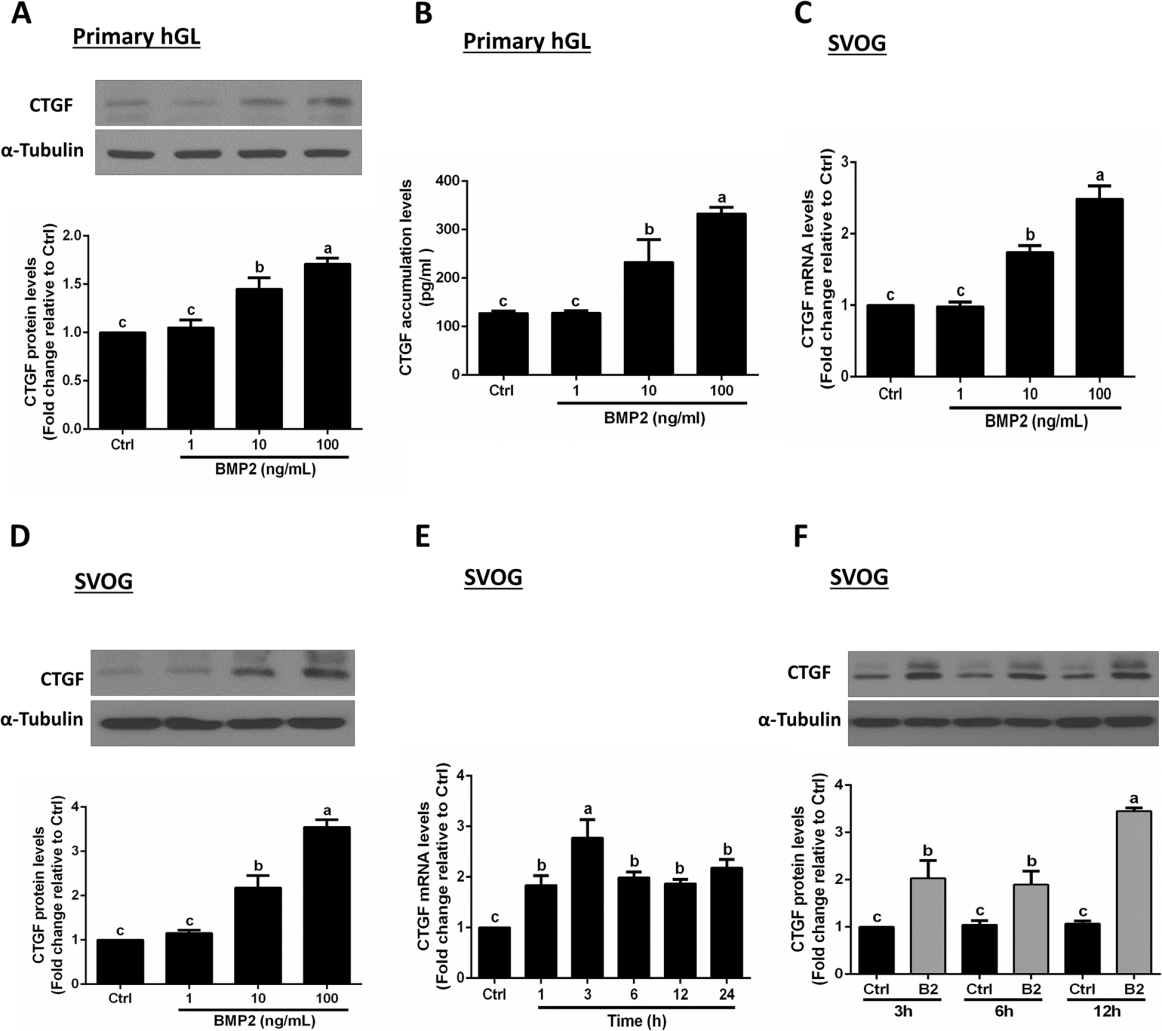
Effects of BMP2 on the expression and accumulation of CTGF in hGL cells. **A** and **B** Primary hGL cells were treated with Ctrl or different concentrations (1, 10, or 100 ng/ml) of BMP2 for 24 h. The protein levels of CTGF were examined using western blot analysis (**A**), and the accumulated levels of CTGF in conditioned medium were examined using ELISA (**B**). **C** and **D** SVOG cells were treated with Ctrl or different concentrations (1, 10 or 100 ng/ml) of BMP2 for 24 h, and the mRNA (**C**) and protein (**D**) levels of CTGF were examined using RT-qPCR and western blot analysis, respectively. **E** and **F** SVOG cells were treated with Ctrl or BMP2 (50 ng/ml) at different time points (0.5, 1, 3, 6, 12 or 24 h), and the mRNA (**E**) and protein (**F**) levels of CTGF were examined using RT-qPCR and western blot analysis, respectively. The results are expressed as the mean ± SEM of at least 3 independent experiments, and values without common letters are significantly different (*P* < 0.05)

Given that CTGF is a critical intraovarian factor that regulates mammalian ovulation, we next investigated whether CTGF is involved in the BMP2-induced increase in hyaluronan production in human granulosa cells. First, the results showed that BMP2 treatment significantly increased the protein levels of CTGF in a dose-dependent manner in primary hGL cells (Fig. 2A). In addition, we confirmed that BMP2 increased the accumulated levels of CTGF in the conditioned culture medium (Fig. 2B). Similarly, using SVOG cells, the results showed that BMP2 significantly increased the mRNA and protein levels of CTGF in a concentration-dependent manner (Fig. 2C and D). The time course studies showed that BMP2 increased the mRNA and protein levels of CTGF, starting at 1 h and 3 h, respectively (Fig. 2E and F).

### CTGF is a mediator that contributes to BMP2-induced upregulation of HAS2 expression and hyaluronan production in hGL cells

**Fig. 3:**
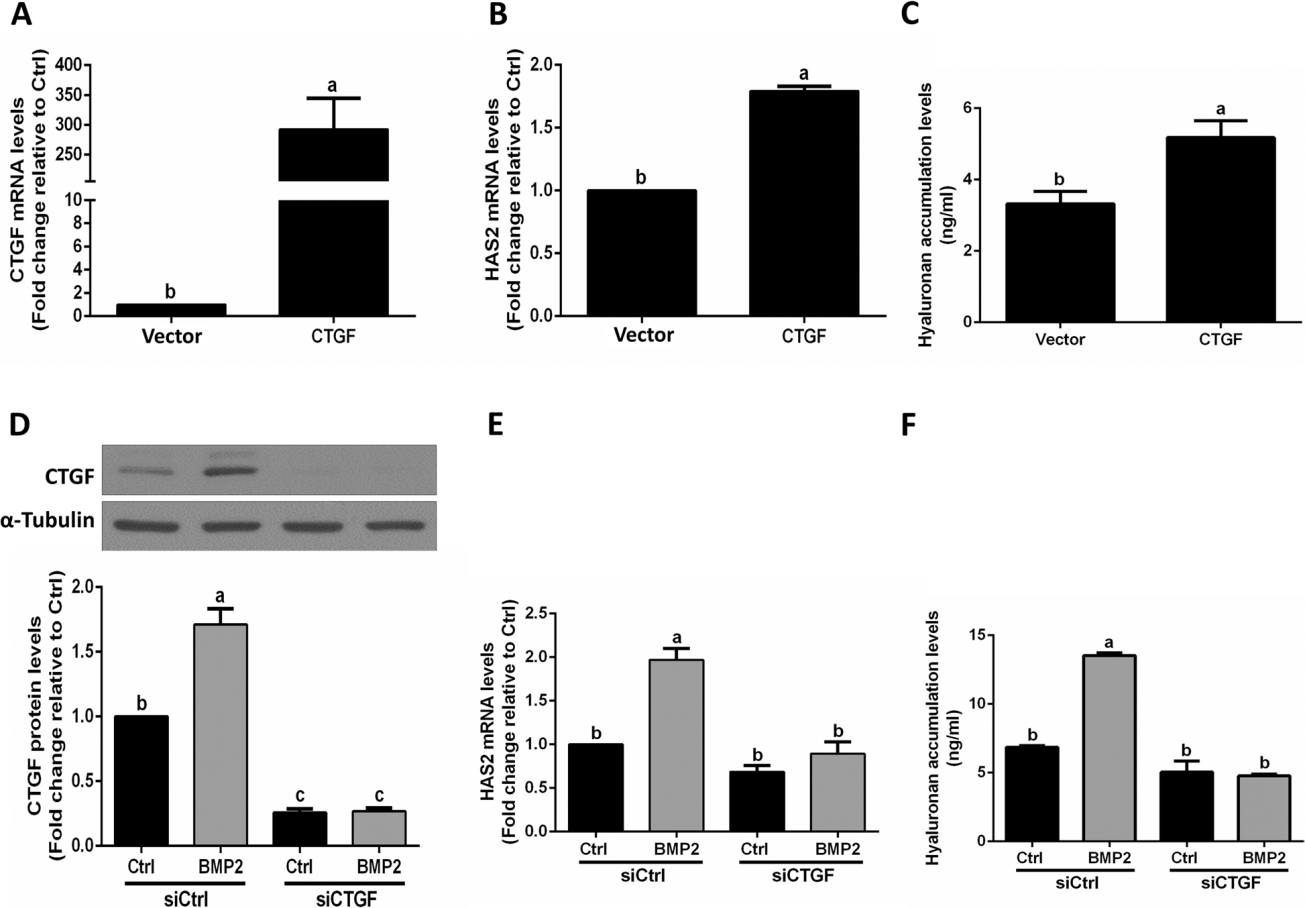
CTGF mediates BMP2-induced upregulation of HAS2 expression and increases hyaluronan synthesis in SVOG cells. **A-C** SVOG cells were transfected with empty vector control (Vector) or CTGF overexpression vector (1 μg, CTGF) for 48 h. The mRNA levels of CTGF (**A**) and HAS2 (**B**) were examined using RT-qPCR, and the accumulated levels of hyaluronan were examined using ELISA (**C**). D-F, SVOG cells were transfected with control siRNA (siCtrl) or siRNA targeting CTGF (siCTGF) for 48 h, and then the cells were treated with Ctrl or BMP2 (50 ng/ml) for an additional 24 h. The protein levels of CTGF were examined using western blot analysis (**D**). The mRNA levels of HAS2 were examined using RT-qPCR (**E**). The accumulated levels of hyaluronan in conditioned medium were examined using ELISA (**F**). The results are expressed as the mean ± SEM of at least 3 independent experiments, and values without common letters are significantly different (*P* < 0.05)

We next investigated whether CTGF plays a role in the BMP2-induced upregulation of HAS2 using stable overexpression in SVOG cells. An empty vector control (Vector) was generated in parallel. Then, CTGF-overexpressing SVOG cells were analyzed by RT-qPCR (Fig. 3A). As shown in Fig. 3B and C, CTGF overexpression induced increased expression of HAS2 and increased the production of hyaluronan in SVOG cells. Furthermore, using a siRNA-based knockdown approach, we demonstrated that the knockdown of endogenous CTGF significantly decreased the protein levels of CTGF in SVG cells (Fig. 3D). Notably, knockdown of CTGF completely reversed the BMP2-induced increase in the mRNA levels of HAS2 and hyaluronan production in SVOG cells (Fig. 3E and F).

### The BMP type I receptor inhibitors DMH-1 and dorsomorphin completely abolish the BMP2-induced upregulation of CTGF in SVOG cells

**Fig. 4:**
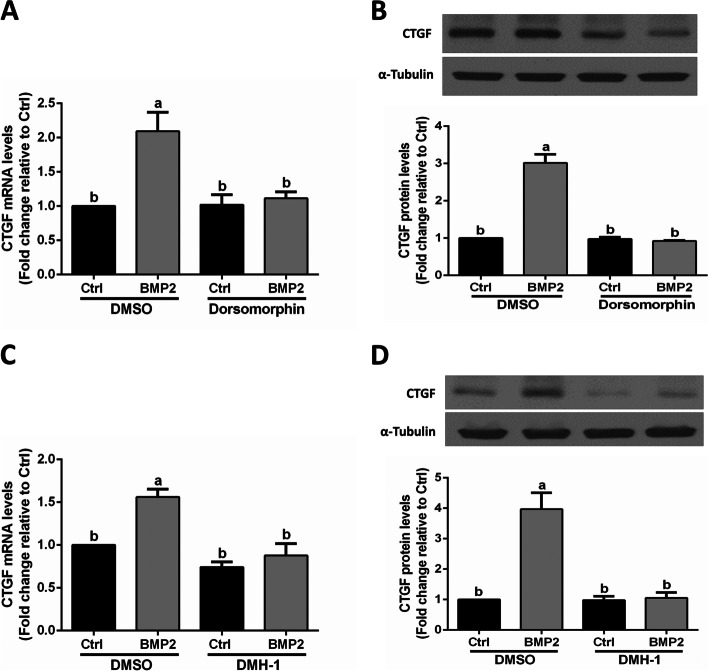
Effect of BMP type I receptor inhibitors on the BMP2-induced upregulation of CTGF expression in SVOG cells. **A** and **B** SVOG cells were pretreated with dorsomorphin (10 μM) for 1 h, and then the cells were treated with Ctrl or BMP2 (50 ng/ml) for an additional 6 h (**A**) or 12 h (**B**). The mRNA (**A**) and protein (**B**) levels of CTGF were examined using RT-qPCR and western blot analysis, respectively. **C** and **D** SVOG cells were pretreated with DMH-1 (0.25 μM) for 1 h, and then the cells were treated with Ctrl or BMP2 (50 ng/ml) for an additional 6 h (**C**) or 12 h (**D**). The mRNA (**C**) and protein (**D**) levels of CTGF were examined using RT-qPCR and western blot analysis, respectively. The results are expressed as the mean ± SEM of at least 3 independent experiments, and values without common letters are significantly different (*P* < 0.05)

To explore which BMP type I receptor is involved in the BMP2-induced upregulation of CTGF expression, SVOG cells were pretreated with two BMP type I receptor inhibitors (DMH-1 and dorsomorphin) followed by BMP2 treatment. Our results showed that DMH-1 (an inhibitor of ALK2 and ALK3) treatment completely abolished the BMP2-induced upregulation of CTGF (at the mRNA and protein levels) in SVOG cells (Fig. 4A and B). Similarly, dorsomorphin (an inhibitor of ALK2, ALK3, and ALK6) treatment completely abolished the BMP2-induced upregulation of CTGF (at the mRNA and protein levels) in SVOG cells (Fig. 4C and D).

### BMP type I receptors ALK2 and ALK3 are involved in mediating the BMP2-induced increase in CTGF expression in SOVG cells

**Fig. 5:**
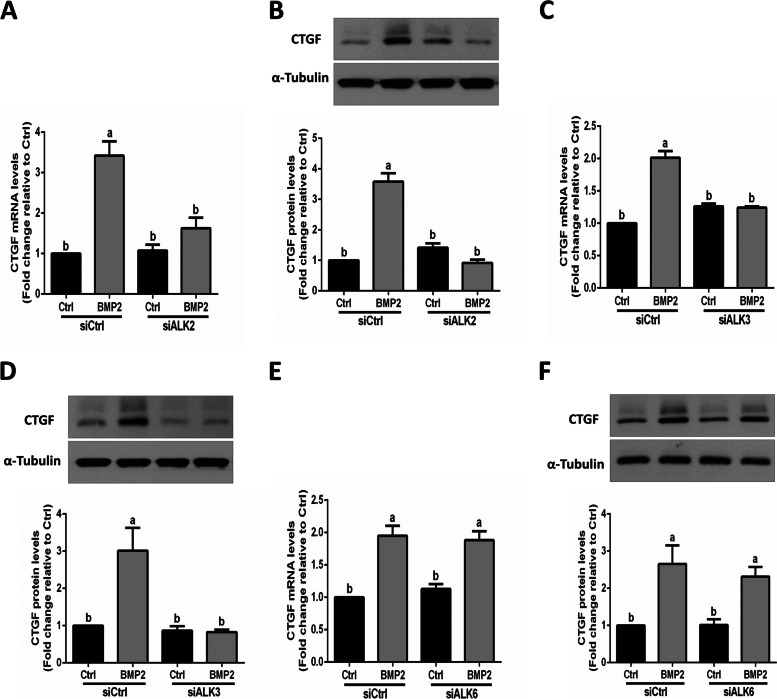
The TGF-β type I receptors ALK2 and ALK3 are involved in the BMP2-induced upregulation of CTGF expression in SVOG cells. **A** and **B** SVOG cells were transfected with siCtrl or siRNA targeting ALK2 (siALK2) for 48 h, and the cells were treated with Ctrl or BMP2 (50 ng/ml) for an additional 6 h (**A**) or 12 h (**B**). The mRNA (**A**) and protein (**B**) levels of CTGF were examined using RT-qPCR and western blot analysis, respectively. **C** and **D** SVOG cells were transfected with siCtrl or siRNA targeting ALK3 (siALK3) for 48 h, and the cells were treated with Ctrl or BMP2 (50 ng/ml) for an additional 6 h (C) or 12 h (**D**). The mRNA (**C**) and protein (**D**) levels of CTGF were examined using RT-qPCR and western blot analysis, respectively. **E** and **F** SVOG cells were transfected with siCtrl or siRNA targeting ALK6 (siALK6) for 48 h, and the cells were treated with Ctrl or BMP2 (50 ng/ml) for an additional 6 h (**E**) or 12 h (**F**). The mRNA (**E**) and protein (**F**) levels of CTGF were examined using RT-qPCR and western blot analysis, respectively. The results are expressed as the mean ± SEM of at least 3 independent experiments, and values without common letters are significantly different (*P* < 0.05)

Considering the off-target effects of kinase inhibitors, we next used a siRNA-mediated approach to confirm which ALK was the mediator that participated in the BMP2-induced increase in CTGF expression in human granulosa cells. Specifically, we knocked down specific BMP type I receptors, including ALK2, ALK3 and ALK6 in SVOG cells. The knockdown results showed that knockdown of either ALK2 or ALK3 completely reversed the BMP2-induced increases in the mRNA and protein levels of CTGF in SVOG cells (Fig. 5A-D). However, knockdown of ALK6 did not have such effects (Fig. 5E and F).

### SMAD signaling mediates the BMP2-induced increases in the expression of CTGF and HAS2 as well as the production of hyaluronan in SVOG cells

**Fig. 6:**
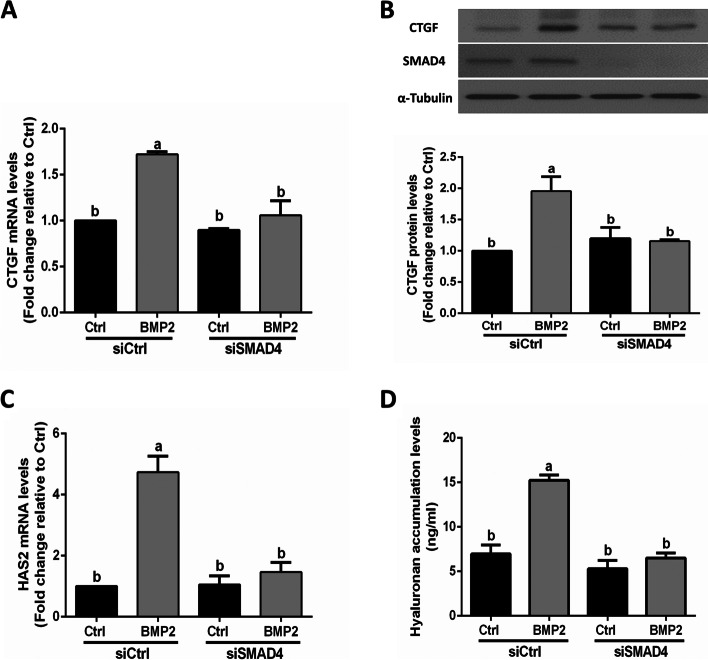
SMAD4 signaling is required for the BMP2-induced upregulation of CTGF expression and the increase in hyaluronan synthesis in SVOG cells. **A** and **B** SVOG cells were transfected with siCtrl or siRNA targeting SMAD4 (siSMAD4) for 48 h, and the cells were treated with Ctrl or BMP2 (50 ng/ml) for an additional 6 h (**A**) or 12 h (**B**). The mRNA (**A**) and protein (**B**) levels of CTGF were examined using RT-qPCR and western blot analysis, respectively. **C** and **D** SVOG cells were transfected with siCtrl or siSMAD4 for 48 h, and the cells were treated with Ctrl or BMP2 (50 ng/ml) for an additional 12 h (**C**) or 24 h (**D**). The mRNA levels of HAS2 were examined using RT-qPCR (**C**), and the accumulated levels of hyaluronan in the conditioned medium were examined using ELISA (**D**). The results are expressed as the mean ± SEM of at least 3 independent experiments, and values without common letters are significantly different (*P* < 0.05)

Upon binding of BMPs with their functional receptors, the downstream R-SMAD proteins are phosphorylated, which in turn associate with the common SMAD protein, SMAD4 to form a trimer complex. This complex finally translocates into the nucleus and regulates the target gene transcription [34]. To explore whether SMAD signaling is involved in the effect of BMP2 on the expression of CTGF and hyaluronan production, SVOG cells were transfected with the specific siRNA targeting SMAD4 to knock down endogenous SMAD4. The results showed that knockdown of SMAD4 completely reversed the BMP2-induced upregulation of CTGF at the mRNA and protein levels (Fig. 6A and B). Notably, knockdown of SMAD4 completely reversed the BMP2-induced increases in the mRNA levels of HAS2 (Fig. 6C) and hyaluronan production (Fig. 6D) in SVOG cells. These findings indicate that SMAD4 signaling is required for the BMP2-induced upregulation of CTGF and HAS2 expression and an increase in hyaluronan production in human granulosa cells.

## Discussion

Successful ovulation heavily relies on the proper expansion of COC under the control of gonadotropin and locally produced growth factors [1, 35]. Increasing evidence suggests that aberrant expression of BMPs and their downstream signaling may cause female infertility [36]. Previous in vitro studies have shown that BMPs promote COC expansion by regulating the expression of several proteins related to COC expansion [37–41]. Previous studies have shown that several BMPs, including BMP2, BMP4, BMP5, BMP6, BMP7 and BMP8A are expressed in the granulosa cells from normally cycling and polycystic ovary syndrome women [24]. In the human corpus luteum, BMP2, BMP4 and BMP6 are highly expressed in the granulosa-lutein and theca-lutein cells and are involved in the process of luteolysis [24]. Moreover, the mature protein of BMP2 can be detected in human follicular fluid (1–115 ng/ml) [36]. We thus chose the granulosa cell-derived BMP2 to investigate the functional role in regulating hyaluronan synthases in this study. In the present study, we demonstrated that BMP2 increased hyaluronan production in hGL cells. These results are consistent with a previous study showing that BMP4, BMP6, BMP7 and BMP15 increase the production of hyaluronan in hGL cells [41]. Additionally, our results showed that BMP2 treatment significantly upregulated the expression of HAS2, but not HAS1 and HAS3, in a dose and time-dependent manner. Similarly, animal studies showed that only HAS2 is upregulated in response to stimulation with gonadotropins, whereas HAS1 is slightly upregulated, and HAS3 is not affected by gonadotropins, indicating that HAS2 is the principal enzyme involved in the production of hyaluronan in hGL cells [42, 43].

Previous studies have shown that several members of the TGF-β superfamily can promote the production of hyaluronan by regulating the expression of HAS2 [41, 44, 45]. However, the precise mechanisms underlying this regulatory activity have not been determined. In the present study, we showed that CTGF mediates the BMP2-induced upregulation of HAS2 expression and hyaluronan synthesis in hGL cells. This conclusion is based on our results showing that BMP2 upregulates the expression of CTGF at the mRNA and protein levels as early as 1 h and 3 h, respectively. However, the mRNA level of HAS2 was not increased until up to 3 h after BMP2 treatment. Most importantly, overexpression of CTGF significantly increased the mRNA level of HAS2 and the production of hyaluronan, and knockdown of CTGF completely abolished the BMP2-induced upregulation of HAS2 expression and hyaluronan production in human granulosa cells. These findings indicate that CTGF is the downstream mediator of BMP2 in promoting hyaluronan production in hGL cells. Given the essential role of CTGF and hyaluronan in COC expansion, our studies further confirm that the modulation effect of BMP2 on CTGF expression and hyaluronan synthesis is an essential cellular mechanism regulating ovulation by modulating proper COC expansion during the late stage of follicle development. Nevertheless, we acknowledge that all of our results were based on in vitro cell experiments, with no evidence showing that BMP2 directly affects follicular COC expansion in vivo. Future animal studies aimed at addressing this issue will be of great interest to support our in vitro findings in the current study.

The activation of the TGF-β superfamily signaling pathway depends on the combination of the ligands and their related receptors. Upon ligand–receptors binding, type II receptors phosphorylate type I receptors to form an activated ligand/receptor complex, which in turn activates the downstream effector proteins, the R-SMADs [34]. Compared with more than forty TGF-β superfamily members, only seven TGF-β type I and five TGF-β type II receptors have been identified. A large number of studies have demonstrated that the interaction modes between the ligands and their corresponding receptors are diverse in different tissues or cell types [46]. Importantly, the mutation or aberrant expression of TGF-β superfamily members or receptors is associated with reproductive disorders [36]. Thus, a detailed exploration of the interaction between ligands and their relevant receptors will help with understanding the underlying molecular mechanisms by which TGF-β superfamily members exert their cellular activities and the related pathological conditions. In general, three TGF-β type I receptors, including ALK2, ALK3 and ALK6, have been reported to bind to specific BMP ligands [34]. In the current study, using pharmacological and siRNA-based knockdown approaches, we demonstrated that ALK2 and ALK3 receptors are involved in the BMP2-induced upregulation of CTGF expression in hGL cells. This result is consistent with our most recent study showing that BMP6 increases the expression of CTGF via both ALK2 and ALK3 receptors [47]. A previous in vivo study demonstrated that the specific TGF-β type I receptor inhibitor dorsomorphin (a selective inhibitor of ALK2/ALK3/ALK6) completely attenuates the inductive effect of BMP2 on primordial follicle formation in mouse ovaries, whereas DMH-1 (a selective inhibitor of ALK2/ALK3) only partially abolishes the inductive effect of BMP2 [25]. In contrast, our study showed that DMH-1 and dorsomorphin both completely abolished the BMP2-induced upregulation of CTGF expression in hGL cells, indicating that ALK2/ALK3 but not ALK6 is involved in the regulatory effect of BMP2. The discrepancy between two studies may explain the complexity of the BMP-receptor interaction, and the combination model of BMPs and their receptors is cell type- or species-dependent. In this regard, only ALK3 but not ALK2 is involved in the regulatory effect of BMP2 on cell invasion in human trophoblast cells [48]. Collectively, these findings suggest that the activation of the BMP2 signaling pathway is dependent on specific receptors, which provides a new insight into molecular targets for treating female reproductive disorders.

In the canonical BMP signaling pathway, phosphorylated type I receptors recruit and phosphorylate the downstream R-SMAD proteins, which in turn form trimers with SMAD4 and translocate into the nucleus to regulate target gene expression [34]. Data obtained from in vivo studies showed that granulosa cell-specific *Smad4* knockout mice are subfertile and exhibit multiple defects in follicle development and ovulation, including disrupted steroidogenesis, impaired cumulus function, and premature luteinization [49]. These results highlight the essential role of SMAD4 in follicular function. In this study, using specific siRNA-mediated knockdown approach, we showed that knockdown of endogenous SMAD4 completely reversed the BMP2-induced upregulation of CTGF expression and hyaluronan production, indicating that the SMAD4-derived signaling pathway is necessary for the regulatory effect of BMP2 on hyaluronan synthesis. Although we did not provide data showing that SMAD1/5/8 is involved in the BMP2-induced hyaluronan synthesis, our previous study demonstrated that BMP2 treatment induced the phosphorylation of SMAD1/5/8 in hGL cells and that BMP2 regulated its target gene expression via the SMAD1/5/8-SMAD4 signaling pathway [27, 50]. Taken together, our results suggest that the BMP2-induced upregulation of CTGF expression and hyaluronan synthesis is most likely mediated by the SMAD1/5/8-SMAD4 signaling pathway in hGL cells.

**Fig. 7:**
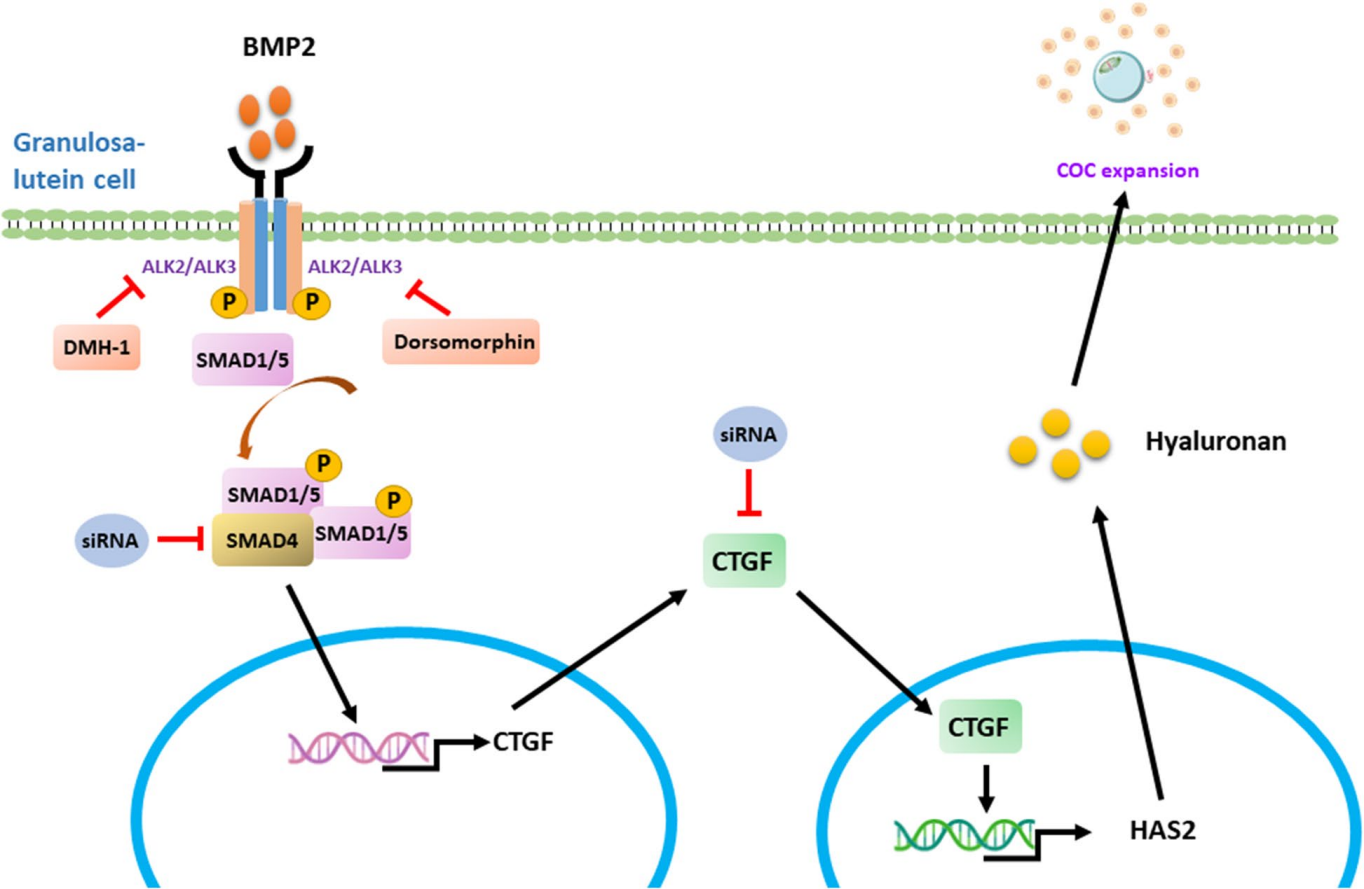
Schematic diagram illustrating the proposed molecular mechanism by which BMP2 increases the production of hyaluronan in hGL cells. The BMP2 ligand binds to BMP type I and type II receptors located on the cell membrane of hGL cells, which then phosphorylates the downstream receptor-regulated SMAD SMAD1/5. The phosphorylated SMAD1/5 combine with the common SMAD SMAD4 to form a heterotrimeric transcription factor complex, which subsequently translocates into the nucleus and upregulates the expression of CTGF and HAS2 as well as the production of hyaluronan in hGL cells. CTGF is the regulator that mediates the BMP2-induced upregulation of HAS2 expression and hyaluronan synthesis in hGL cells. P, phosphorylated

In conclusion, we have demonstrated that BMP2 upregulates the expression of CTGF and HAS2, which in turn increases the production of hyaluronan in hGL cells. Overexpression and siRNA-mediated knockdown results showed that CTGF is the regulator that mediates the BMP2-induced upregulation of HAS2 expression and hyaluronan synthesis in hGL cells. Additionally, both DMH-1 and dorsomorphin abolish the BMP2-induced upregulation of CTGF expression. Furthermore, ALK2 and ALK3, but not ALK6, are the functional receptors required for the inductive effect of BMP2 on CTGF expression (Fig. 7). Taken together, our findings suggest that intraovarian BMP2 plays a vital role in regulating ovulation by modulating the production of hyaluronan in human granulosa cells.

## Data Availability

All data generated or analyzed during this study are included in this published article.
